# Transgenerational effect of Afidopyropen on *Bemisia tabaci* Gennadius (Homoptera: Aleyrodidae)

**DOI:** 10.1038/s41598-023-46479-0

**Published:** 2023-11-15

**Authors:** Muhammad Salman Shafi, Naeem Iqbal, Muhammad Nadir Naqqash, Shafqat Saeed, Muhammad Usman, Allah Ditta Abid, Muhammad Sohail Shahzad, Hasan Riaz, Muhammad Amjad Bashir, Reem Atalla Alajmi

**Affiliations:** 1 Institute of Plant Protection, MNS University of Agriculture, Multan, Pakistan; 2 Department of Agronomy, MNS University of Agriculture, Multan, Pakistan; 3 Department of Plant Protection, Ministry of National Food Security & Research, Karachi, Pakistan; 4 Department of Plant Protection, Faculty of Agricultural Sciences, Ghazi University, Dera Ghazi Khan, Punjab Pakistan; 5 College of Plant Protection, South West University, Chongqing, China; 6 Department of Zoology, Faculty of Science, King Saud University, 11451 Riyadh, Saudi Arabia

**Keywords:** Agroecology, Ecological modelling

## Abstract

*Bemisia tabaci* Gennadius (Homoptera: Aleyrodidae) is the most devastating insect-pest in cotton crop. It is vector of the cotton leaf curl virus (CLCV) and is responsible for huge losses to cotton industry in Pakistan and worldwide. It is mainly controlled by insecticides but the injudicious use of insecticides has resulted in insecticide resistance and population resurgence in addition to various harmful effects on the humans, non-target organisms and the environment. Transgenerational studies are very helpful to choose a best insecticidal option. In the current study, age-stage two-sex life table analysis was used to identify transgenerational effects of sublethal doses of afidopyropen. The adults of *B. tabaci* were treated with three concentrations of afidopyropen i.e., LC_10_, LC_30_ and LC_50_. The results indicated significant changes in the progeny i.e. the fecundity decreased in treated population; and female and male longevity of their progeny were more in control as compared to treated populations. Similarly, population parameters like intrinsic rate of growth (*r*), net reproductive rate (*R*_0_) and limiting rate of growth (*λ*) were significantly decreased in the treated adult progeny with values of 0.08–0.11, 4.85–7.46 and 1.09–1.12 per day, respectively. Based on the reduced biotic potential, afidopyropen can be suggested as an effective alternative option for the management of *B. tabaci*.

## Introduction

The cotton, *Gossypium hirsutum* L. (Malvaceae) is an important fibre and cash crop of tropical and sub-tropical countries with an annual economic impact of about $600 billion worldwide^1^. The cotton crop is attacked by different insect pests which decrease the yield by 30–40% annually^2^. The whitefly, *Bemisia tabaci* Gennadius (Homoptera: Aleyrodidae) is one of the most devastating and challenging insect pests of cotton. It is highly polyphagous pest and can feed on more than 600 distinct plant species. It damages the plants both directly by sucking the phloem sap and indirectly by spreading begomoviruses. Additionally, it secretes honey dew which promotes the sooty mould growth on plant leaves and thus reduces the photosynthetic activity of plants^3^.

Insecticides are considered as an important and effective tool for managing insect pests in different crops^4,5^. Despite the many advantages of insecticides, their widespread and injudicious usage has resulted in serious harmful effects to humans and the environment^6^. Afidopyropen derived from the opportunistic fungal pathogen, *Aspergillus fumigatus* is very effective for the management of whiteflies^7^. The transient receptor potential vanilloid (TRPV) of insects is the target of this novel pyropene pesticide. It interfers with the feeding and other behavior leading to starvation and death of target insect^8^.

Traditional bioassays do not provide full information about the usage of pesticides over an extended period of time^9^. Insecticides should be thoroughly examined as a potential pest control strategy through transgenerational studies on the progeny of insects^10^. Physiological and life table parameters, feeding behaviour, fertility, and several other physical and genetic traits are affected by the sublethal effects of various insecticides^11^. For instance, hatching efficiency, eggs size, and reproductive biology in diamondback moth decreased at sublethal levels of spinosad^12^.

Certain insecticides such as imidacloprid, bifenthrin, buprofezin and cycloxaprid have been shown to have sublethal effects on whiteflies. Some of these effects include delayed development time and a decrease in the fertility of the F_1_ generation^13^. Afidopyropen has recently been introduced and registered in Pakistan for the management of aphids, whitefly and jassid. For this purpose, a thorough evaluation of the transgenerational effects of afidopyropen on *B. tabaci* must be carried out in addition to examining its impact on mortality of *B. tabaci*^14^. In the current study, sublethal effects of afidopyropen were examined on several life table parameters by using the Age-stage, Two-sex life table. For better comparison, a life table was constructed using the offspring of populations of exposed and unexposed *B. tabaci*.

## Materials and methods

### Rearing of *B. tabaci*

*B. tabaci* population was reared using methodology of Esmaeily et al.^15^ with some modifications. The commercially available cotton cultivar (MNH-1050) was obtained from the Cotton Research Institute, Multan, Pakistan and sown in earthen pots (15 L volume) in greenhouse of MNS University of Agriculture, Multan, Pakistan. These plant were maintained under standard agronomic procedures and covered with net mesh bag (60 × 60 × 60 cm) to prevent pest infestation. The adults’ *B. tabaci* were released on these plants after 30 days of sowing to obtain whitefly culture for further experimentation.

### Insecticide

The insecticide, afidopyropen (Sefina™ 5% DC, manufactured and formulated by BASF Corporation, USA; imported by BASF Pakistan Pvt. Ltd. and marketed by Engro Fertilizers Limited, Pakistan) was purchased from the local market in Multan, Pakistan.

### Lethal concentration estimation

The adult whitefly culture maintained in the greenhouse was used for laboratory studies. For this purpose, plants were grown in plastic pots (each measuring 15 × 45 × 15 cm). The bioassays were carried out using the methodology of Heydari et al.^16^ with little modification. About 14 days old plants were selected and placed in meshed cages in laboratory at photoperiod of 14:10 (L:D), 35 ± 5 °C temperature and 60 ± 5% relative humidity. Six concentration (causing 0–100% mortality) of afidopyropen were prepared in distilled water. The leaves were dipped in a specific concentration of insecticide for 20 s and dried for five minutes before releasing *B. tabaci* on plants. There were four plants (replication) for each concentration. Each treated plant was placed in a two-sided mesh plastic cages (measuring 15 × 15 × 15 cm) and10 adult *B. tabaci* were released on each plant. The mortality data was recorded after 72 h of exposure.

### Sublethal effects of afidopyropen on *B. tabaci*

For this experiment, LC_50_, LC_30_, and LC_10_ concentrations of afidopyropen calculated from above bioassay study were used. About 3 weeks old plants with 4–5 leaves were selected and treated with specific concentrations using the methodology described for bioassay study. Each treated plant was placed in meshed cage and 40 adult *B. tabaci* were released into the cage for feeding on treated plants for 1 day. After 1 day, the treated plants were replaced with untreated plants. The adult whiteflies allowed to feed and breed on these untreated plants till end of the experiment^15^. The number of offspring produced by females, the length of oviposition cycles, and other population parameters were monitored daily.

Following the treatment of F_0_, the adult survival and the number of eggs that each female laid everyday were recorded until their death. The survival rate, longevity of various life stages, and adult emergence were also observed. The conditions for transgenerational experiments were 35 ± 5 °C temperature, 60 ± 5% relative humidity and 14:10 (L:D) photoperiod. Following the arrangement and evaluation of the findings, various lethal concentration levels were determined to construct Age-stage, Two-sex life table.

### Life table analysis

Raw data on life table parameters were analysed according to Chi and Su^17^. The main life-table parameters that were calculated include age-stage survival rate (*S*_*xj*_), age-specific survival rate (*l*_*x*_), probability of a newly laid egg surviving to age x, female age-specific fecundity (*f*_*x*_), age-specific fecundity (*m*_*x*_), mean fecundity of individuals at age x, age-specific maternity (*l*_*x*_*m*_*x*_), and age-stage life expectancy. The TWOSEX-MS Chart programme was used to determine demographic characteristics such as the net reproduction rate (*R*_0_), intrinsic rate of increase (*r*), finite rate of rise (*λ*), and mean generation time (*T*). With 100,000 bootstraps, we estimated the variances, standard errors, and means using the bootstrap technique (100,000 bootstraps produced less variable findings and a normal frequency distribution, which were not influenced by the difference in sample sizes). Paired bootstrap test was used to identify the results with significant differences^18–22^.

### Ethical statement

All methods related to plants were conducted in accordance with relevant institutional and national guidelines in the “Materials and methods” section.

## Results

### Lethal concentration estimation

The population of *B. tabaci* was exposed to different concentrations of afidopyropen and the LC_10_, LC_30_ and LC_50_ values were calculated based on the mortality data collected after 72 h of exposure. The LC_10_, LC_30_ and LC_50_ values for the afidopyropen were 11.66, 74.69 and 270.25 µg/mL while the chi-square (χ^2^) value was 3.29 (Table 1).

**Table 1:** Lethal concentration estimation of afidopyropen against whitefly after 72 h.

Insecticide	LC_10_ (95% FL) (µg mL^−1^)	LC_30_ (95% FL) (µg mL^−1^)	LC_50_ (95% FL) (µg mL^−1^)	χ^2^	DF	Slope
Afidopyropen	11.66 (2.432–2.759)	74.69 (45.038–137.104)	270.25 (144.924–1115.871)	3.29	20	0.939 ± 0.221

*LC* lethal concentration, *DF* degree of freedom, χ^2^ Chi square.

### Survival and fecundity of parental generation

**Figure 1:**
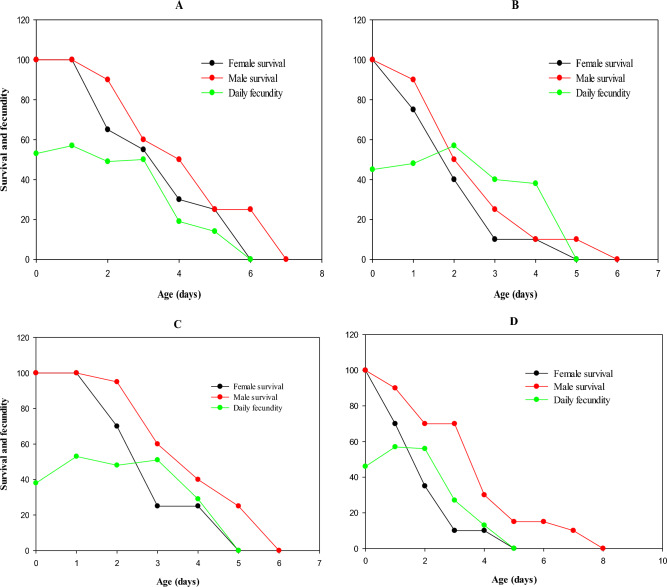
Sublethal effect of different concentrations (**A**) control, (**B**) LC_10_, (**C**) LC_30_, and (**D**) LC_50_ on survival rate and fecundity of parental generation (F_0_) of *Bemisia tabaci.*

Higher male survival rate was observed in the LC_50_-treated population, while the lower male survival rate was observed in LC_30_ and LC_10_-treated population of *B. tabaci*. Survival rate of females was higher in the control, and lower female survival was observed in all populations treated at LC_10_, LC_30_ and LC_50_ values. Daily fecundity ranged from 13 eggs/day in the LC_50_-treated population while higher fecundity (57 eggs/day) was observed in control, LC_30_ and LC_10_-treated population (Fig. 1).

### Life table parameters of progeny of treated adults

The effects of sublethal doses of afidopyropen on the *B. tabaci* population are presented in Table 2. Significant differences were recorded in different life table parameters i.e. pre-adult duration, female longevity, male longevity, oviposition period, total pre-oviposition period (TPOP), and fecundity (per female) in the progeny of whitefly individuals exposed to different concentrations (LC_10_, LC_30_, and LC_50_) and the control. Pre-adult duration was significantly higher (15.1 days) in the control population followed by populations treated with LC_10_ (14.96 days), LC_30_ (14.72 days) and LC_50_ (13.83 days) values. The female longevity was also higher in the control population (19.8 days) while it was the lowest in LC_50_-treated population (17.92 days). Similarly, male longevity was significantly higher in the control group (20.66 days) while significantly lower in LC_50_-treated population (18.04 days).

**Table 2:** Life table parameters of whitefly against afidopyropen.

Parameters	Control	LC_10_	LC_30_	LC_50_
Pre-adult duration (days)	15.10 ± 0.18a	14.96 ± 0.11a	14.72 ± 0.12a	13.83 ± 0.11b
Female longevity (days)	19.8 ± 0.39a	19.10 ± 0.45a	18.56 ± 0.31a	17.92 ± 0.35b
Male longevity (days)	20.66 ± 0.58a	19.52 ± 0.50a	19 ± 0.44a	18.04 ± 0.44b
APOP (days)	0.76 ± 0.11a	0.75 ± 0.11a	0.81 ± 0.09a	0.73 ± 0.09a
TPOP (days)	15.35 ± 0.14a	15.21 ± 0.09a	15.12 ± 0.08a	15.24 ± 0.08b
Oviposition days	3.85 ± 0.20a	3.42 ± 0.28a	3.24 ± 0.19a	3.12 ± 0.17b
Fecundity (per female)	16.29 ± 1.75a	15.83 ± 2.18a	14.59 ± 1.11a	11.76 ± 1.20b

*APOP* adult pre-oviposition period, *TPOP* total pre-oviposition period of female counted from birth.

Means in adjacent rows with different letters differ significantly (P < 0.05) by bootstrap test use.

The adult pre-oviposition period (APOP) in the control was 0.47 days while in the LC_50_-treated population, APOP was 0.18 days. The total pre-oviposition period (TPOP) ranged from 15.24 to 15.35 days in the treated and control populations. the population in the control group had a higher value for oviposition days (3.85 days) while it was significantly lower in the LC_50_-treated population (3.12 days). The fecundity of *B. tabaci* was also significantly higher in the population that was taken as the control (16.29 eggs/female), as compared to the LC_50_-treated population (11.76 eggs/female).

### Population parameter

The population parameters of *B. tabaci* in different treatments are shown in Table 3. There was non-significant difference among the values of intrinsic rate of increase (*r*), net reproductive rate (*R*_*o*_) and limiting rate of growth (*λ*) while there was a significant difference in the values of mean generation time (*T*) in the progeny of *B. tabaci* individuals exposed to different concentrations (LC_10_, LC_30_, and LC_50_) and control. The intrinsic rate of increase (*r*) in the control was 0.11 per day while it was 0.08 per day in the LC_50_-treated population. Similarly, the net reproductive rate (*R*_*o*_) was also higher in the control group (7.46 offsprings/day) as compared to (LC_50_-treated population (4.85 offsprings/day). The mean generation time (*T*) ranged from 17.36 to 18.08 days in the progeny of the LC_50_-treated and control population. The limiting rate of growth (*ʎ*) was relatively higher in the control group (1.12/day) than in the LC_50_-treated population (1.09/day). Based on population parameters, afidopyropen has no major effect on the next generation of a population that has been treated.

**Table 3:** Population parameters of whitefly against afidopyropen.

Parameters	Control	LC_10_	LC_30_	LC_50_
*r* (per day)	0.11 ± 0.01a	0.10 ± 0.01a	0.10 ± 0.01a	0.08 ± 0.01a
*R*_*o*_ (per day)	7.46 ± 1.48a	6.86 ± 1.49a	6.48 ± 1.18a	4.85 ± 0.98a
*T*	18.08 ± 0.17a	17.81 ± 0.18a	17.66 ± 0.14a	17.36 ± 0.21b
*λ* (per day)	1.12 ± 0.01a	1.11 ± 0.01a	1.10 ± 0.07a	1.09 ± 0.01a

*r* intrinsic rate of growth,* R*_0_ the net reproductive rate (offspring/individual), *T* the mean generation time (days), *λ* limiting rate of growth.

Means in adjacent rows with different letters differ significantly (P < 0.05) by bootstrap test use.

### Age-stage specific survival rate (*S*_*xj*_)

**Figure 2:**
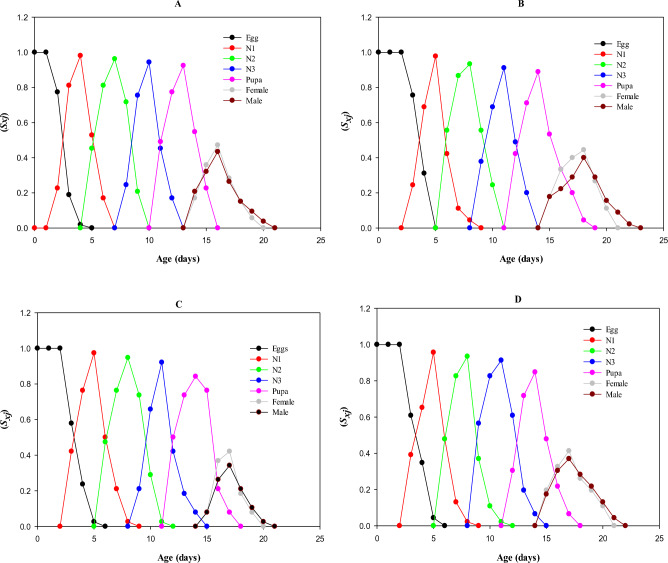
Transgenerational effect of afidopyropen, (**A**) control, (**B**) LC_10_, (**C**) LC_30_, and (**D**) LC_50_ on Age stage specific survival rate (*S*_*xj*_) of *Bemisia tabaci.*

Females in the control group had more age-stage specific survival rate (0.47) as compared to males (0.43) on day 16. On 18th day of LC_10_-treated population, the *S*_*xj*_ values in adult females and males peaked at 0.44 and 0.40, respectively. On 17th day, the males in LC_30_-treated group had *S*_*xj*_ values of around 0.42, whereas females in the same group had values of about 0.34. On 17th day of LC_50_-treated population, female and male adults had age-stage specific survival rate of 0.41 and 0.36, respectively. On day 21st, the *S*_*xj*_ in females dropped to zero, whereas on day 23rd, the same was true for males. Males exposed to LC_30_ had the lowest peak in *S*_*xj*_ (Fig. 2).

### Age-stage life expectancy (*e*_*xj*_)

**Figure 3:**
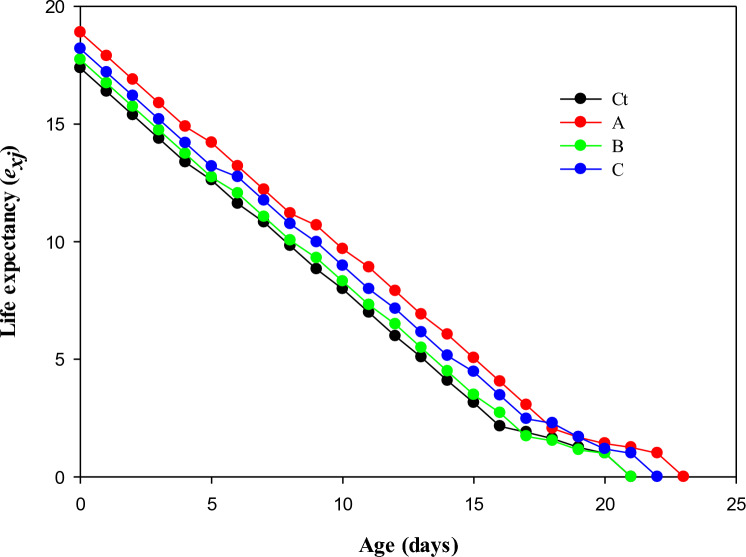
Transgenerational effect of afidopyropen on life expectancy *(e*_*xj*_*)* of *Bemisia tabaci*, (Ct) control, (A) LC_10_, (B) LC_30_, and (C) LC_50_.

The age-stage specific expectancy (*e*_*xj*_) represents the typical lifespan of a species or individual at age *x* and developmental stage *j* (Fig. 3). Values for *e*_*xj*_ were greater in the populations treated with LC_10_ (reaching 0 on the 23rd day) compared to those in control (reaching 0 on the 21st day).

### Age-specific maternity (*l*_*x*_*m*_*x*_)

**Figure 4:**
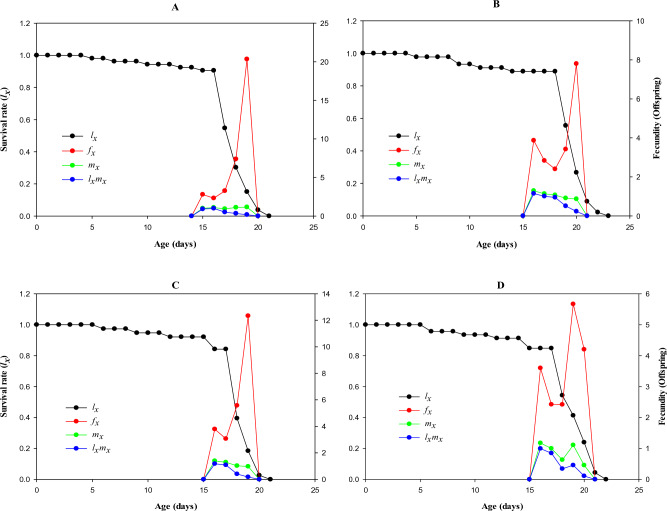
Transgenerational effect of afidopyropen, (**A**) control, (**B**) LC_10_, (**C**) LC_30_, and (**D**) LC_50_ on Age-specific survival rate (*l*_*x*_), age-specific fecundity of total population (*m*_*x*_), and age-specific maternity (*l*_*x*_*m*_*x*_) of initial *Bemisia tabaci* exposed to LC_10_, LC_30_ and LC_50_ of afidopyropen.

Age-specific maternity is another key indicator of population dynamics. A combination of the age-specific survival rate (*l*_*x*_) and the age-specific fecundity (*m*_*x*_) of the entire population determines the age-specific maternity (*l*_*x*_*m*_*x*_). The sum of these characteristics is depicted graphically in Fig. 4. Adults treated with LC_50_ had the lowest age-stage specific fecundity (*f*_*x*_), with just 5.66 offspring at 19 days of age compared to 20.33 offspring from the control treatment.

### Age-stage reproductive values (*v*_*xj*_)

**Figure 5:**
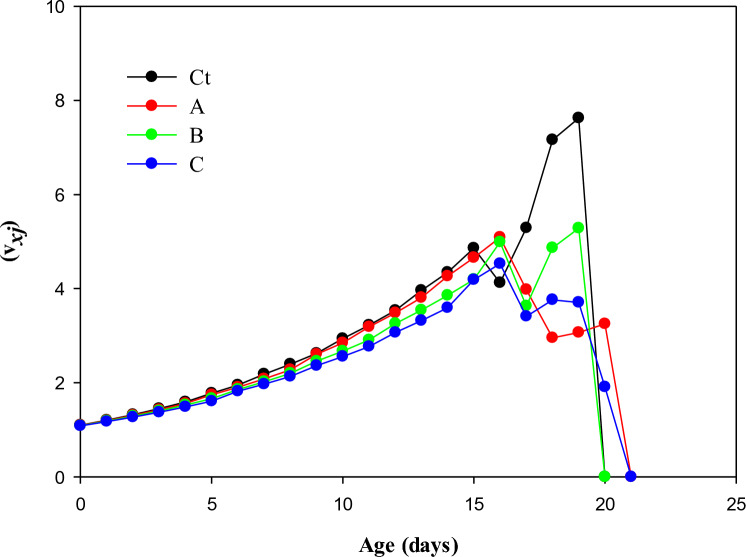
Transgenerational effect of afidopyropen on reproductive value (*V*_*xj*_) on *Bemisia tabaci*, (Ct) control, (A) LC_10_, (B) LC_30_, and (C) LC_50_.

Reproductive age (*v*_*xj*_) has been shown in Fig. 5. On day 19th, the *v*_*xj*_ value was 7.62 for the control population which dropped to 0.0 on day 20. Age-stage reproductive value in the LC_50_-treated population was 4.53 on the 16th day and dropped to zero by the 21st day.

## Discussion

Afidopyropen is a recently launched chemical in Pakistan that could be used to protect crops from sucking insect pests especially whitefly, jassid and aphids^23^. For designing effective pest management strategy, it is crucial to understand the impacts of any pesticide^24,25^. With its high toxicity against *B. tabaci* and lack of cross-resistance, afidopyropen has been reported very effective even against resistant population of *B. tabaci*^26^. It was shown that sublethal concentrations of insecticides affect survival and growth (shortened oviposition period, decreased fecundity, and decreased hatchability of eggs) of insects.

Afidopyropen has been documented to have negative effects on insect pests^14^. Fecundity was relatively lower in the progeny of the treated population as compared to the *B. tabaci* population in control. These results are comparable to the findings of other researchers who have reported that afidopyropen reduce the fecundity of aphid^27,28^. The female longevity was also higher in the control population compared to the treated population. The intrinsic rate of increase (*r*), net reproductive rate (*R*_*o*_), limiting rate of growth (*λ*) and age-stage specific survival rate (*S*_*xj*_) were lower in the treated progeny of *B. tabaci*. These results are similar to the findings of Tang et al*.*^28^, who reported that afidopyropen decreased the values of female longevity, *r*, *R*_*o*_, *λ*, and the age-stage specific survival rate (*S*_*xj*_) of aphids. Sublethal doses of afidopyropen showed a significant decrease in vitellogenins (*Vg*) expression compared to the control group, as revealed by Liu et al.^29^. Overexpression of *Vg* and its receptors *VgR* in insects may lead to enhanced fertility. Additionally, different population parameters viz *r*, *R*_*o*_, and *λ*, were decreased in the afidopyropen-treated population. Inhibitory effects such as lower fecundity, delayed growth, shortened lifespan, impaired motility, or learning, have traditionally been the focus of research on insects’ reactions to sublethal dosages of insecticides^30^. It can be attributed to the fact that afidopyropen has the ability to inhibit the activity of acylCoA: cholesterol acyltransferase^31^. Additionally, afidopyropen targets the transient receptor potential vanilloid (TRPV) of insects, which results in starvation, desiccation, and mortality^32^.

Mean generation time (*T*) was increased in control and decreased in insecticide-treated populations. While Liu et al.^29^ reported that *T* was increased in insecticide-treated populations in aphids. It may be due to the difference in biology of whitefly and aphid i.e. aphids reproduce by parthenogenesis while whitefly is an oviparous insect. The age-stage reproductive (*V*_*xj*_) was higher in the control as compared to other treated populations. Age-specific maternity (*l*_*x*_*m*_*x*_) age-specific survival rate (*l*_*x*_), the overall population’s age-specific fecundity (*m*_*x*_) and age-stage specific fecundity (*f*_*x*_) were relatively lower in the control group as compared to the treated population. These results are comparable with the results of Esmaeily et al.^15^, who reported that *l*_*x*_*m*_*x,*_* l*_*x,*_* m*_*x*_ and *f*_*x*_ are increased under the sublethal concentration of pymetrozin against whiteflies. Nevertheless, it is known that stress had a profound impact on the population dynamics of insects. Insects may adapt by increasing their number of moults or moult duration, developmental time, and reproductive rate in response to these factors. Insects’ immunological reactions, including melanization, lysozyme levels, and phenoloxidase (PO), can be responsible for the alterations in physiology and morphology they exhibit in response to food, gases, and chemicals^33^. Afidopyropen is known to enhance glutathione s-transferases and P450 enzymes in the insects^25, 34^, which in documented to exert significant fitness cost in the afidopyropen-exposed whitefly population as compared to the control^34^.

## Conclusion

Afidopyropen is an effective insecticide for the control of whiteflies. By examining the many factors during this experiment, it is indicated that this insecticide can be quite helpful population suppressor. To control this insect pest, it might be included in an integrated pest management program.

## Data Availability

Datasets used and/or analysed during the current investigation are available from the corresponding author upon reasonable request.
